# A YafK family protein from Legionella pneumophila exhibits serine-dependent β-lactam hydrolysis activity

**DOI:** 10.1016/j.jbc.2026.113293

**Published:** 2026-06-23

**Authors:** Wenwen Xu, Yuan Cao, Liangsan Cao, Changyong Xiao, Wenjie Sun, Zhenhuang Ge, Weiqiang Wang, Jiajia Gao, Honghua Ge

**Affiliations:** 1 School of Life Sciences and Medical Engineering, Anhui University, Hefei, P. R. China; 2 School of Life Sciences, Sun Yat-Sen University, Guangzhou, P. R. China

**Keywords:** antibiotic resistance, cell wall, gram-negative bacteria, Legionella pneumophila, protein structure, X-ray crystallography, YafK protein, β-lactam hydrolysis

## Abstract

Members of the YafK subfamily within the YkuD superfamily have been redefined as cysteine-dependent amide hydrolases involved in cell envelope remodeling. However, whether this conserved scaffold supports additional catalytic chemistries remains unclear. Here, we report the structural and functional characterization of Lpg1514 from *Legionella pneumophila*. Crystal structures show that Lpg1514 adopts a canonical YafK-like fold with a well-defined substrate-binding pocket. Biochemical analysis reveals that Lpg1514 mediates β-lactam turnover. Unexpectedly, this activity depends on a conserved serine residue (Ser132), whereas the canonical cysteine (Cys174) is not required for catalysis under the conditions tested. Mutational and kinetic analysis support a noncanonical serine-dependent mechanism distinct from both YafK-type amidases and cysteine-dependent L, D-transpeptidases. Functionally, Lpg1514 contributes to β-lactam tolerance in *L. pneumophila* in a Ser132-dependent manner, consistent with limited catalytic efficiency and a role in transient antibiotic interaction rather than productive hydrolysis. Together, these findings expand the functional repertoire of the YafK scaffold and demonstrate that this fold can accommodate mechanistically distinct chemistries. The emergence of serine-dependent β-lactam reactivity within a cysteine-centered lineage highlights the catalytic plasticity of the YkuD/YafK family and suggests a potential route for the diversification of antibiotic-interacting functions.

β-Lactam antibiotics inhibit bacterial cell wall synthesis by targeting penicillin-binding proteins (PBPs), thereby blocking the formation of 4→3 peptidoglycan cross-links (1, 2). Bacteria have evolved multiple strategies to tolerate or resist β-lactams, including the production of β-lactamases and the use of alternative cell wall–modifying enzymes (3, 4, 5). Among these, members of the YkuD superfamily, also known as L,D-transpeptidases (LDTs), catalyze 3–3 cross-linking of peptidoglycan and interact with β-lactam antibiotics *via* a catalytic cysteine residue (6, 7, 8, 9).

YafK proteins, a subgroup within the YkuD superfamily, have recently been reassigned as cysteine-dependent amide hydrolases that detach Braun’s lipoprotein from peptidoglycan (10, 11, 12). This functional redefinition has established a conserved catalytic role for the active-site cysteine within this scaffold. However, whether YafK proteins are strictly limited to this cysteine-dependent chemistry or can support alternative catalytic activities remains unknown. Members of the YkuD superfamily, particularly L,D-transpeptidases, are known to interact with β-lactam antibiotics through a catalytic cysteine (13, 14, 15). In contrast, it is unclear whether YafK proteins retain any capacity to engage β-lactam substrates, or whether such interactions, if present, would follow canonical cysteine-dependent mechanisms.

The protein Lpg1514 from *Legionella pneumophila*, annotated as a member of the YafK family, has not been thoroughly characterized for its biochemical properties or physiological role. In this study, we present the structural and enzymatic characterization of Lpg1514. We determined high-resolution crystal structures of Lpg1514ΔN29 and its cysteine mutant, revealing that Lpg1514 exhibits measurable β-lactam hydrolysis activity. Surprisingly, this activity is independent of the conserved cysteine and instead requires a conserved serine residue, with a nearby histidine residue contributing to its activity. Additionally, we show that Lpg1514 contributes to β-lactam tolerance in *L. pneumophila* in a serine-dependent manner. These findings uncover a β-lactam–interacting activity within a YafK protein that differs mechanistically from previously characterized reactions in this family.

## Results

### Overall structure of Lpg1514

To elucidate the structural basis of Lpg1514 function, we determined the crystal structures of an N-terminally truncated construct lacking the predicted lipoprotein signal peptide (Lpg1514ΔN29) and its cysteine mutant, Lpg1514ΔN29^C174A^, at resolutions of 1.5 Å and 2.1 Å, respectively (Table 1). The two structures are nearly identical, with no discernible conformational differences. In both crystals, the asymmetric unit contains six molecules that superimpose closely, with Cα root-mean-square deviation (RMSD) of less than 0.25 Å. Unless otherwise stated, subsequent analyses are based on the higher-resolution Lpg1514ΔN29 structure.

**Table 1:** Data collection and refinement statistics

Parameter	Lpg1514pg1514ΔN29	Lpg1514ΔN29^C174A^
Data collection		
SSRF beamline	BL17U	BL10U2
Wavelength(Å)	0.97861	0.97923
Space group	*P*2_1_	*P*2_1_
Molecules/ASU	6	6
Cell parameters		
a/b/c (Å)	61.44/134.83/99.84	61.49/134.27/99.22
α/β/γ	90.00/104.51/90.00	90.00/104.46/90.00
Resolution range (Å)	48.33–1.55 (1.58–1.55)	48.04–2.10 (2.14–2.10)
No. of unique reflections	225,008 (9770)	81,978 (4943)
*R*_p.i.m._^a^ (%)	6.6 (50.0)	5.5 (28.2)
Average I/σ(I)	10.5 (2.0)	7.7 (1.9)
CC_1/2_	0.994 (0.613)	0.996 (0.808)
Completeness (%)	99.1 (86.1)	90.3 (99.0)
Redundancy	6.7 (5.7)	2.6 (2.7)
Refinement		
PDB entry	25BW	25BY
Resolution limits (Å)	20.0–1.55 (1.61–1.55)	29.8–2.1 (2.18–2.10)
No. of reflections	224,864 (20,862)	81,909 (8939)
R factor^b^ (%)	13.6 (21.1)	15.8 (19.7)
Free R factor^c^ (%)	16.6 (24.6)	19.8 (26.2)
No. of protein atoms	10,137	10,095
No. of ligands	55	55
No. of solvent molecules	1215	791
rmsd^d^ in bond lengths (Å)	0.013	0.008
rmsd in bond angles (°)	1.64	1.08
*B*-factor (Å^2^)		
macromolecules	18.8	32.8
ligands	36.5	63.7
waters	32.4	37.7
Ramachandran plot^e^ (%)		
favored/disallowed	97.45/0	96.74/0

Values in parentheses refer to the highest resolution shell.

a *R*_p.i.m_ = ∑_*hkl*_ [1/(n_*hkl*_ – 1)]^1/2^∑_*i*_ |*I*_*i*_(*hkl*) – ⟨*I*(*hkl*)⟩|/∑_*hkl*_ ∑_*i*_*I*_*i*_(*hkl*), where n_*hkl*_ is the number of observations of reflection *hkl*.

b R-factor = ∑h | |*F*_*obs*_|-|*F*_*calc*_| |/∑|*F*_*obs*_|, where |*F*_*obs*_| and |*F*_*calc*_| are the observed and calculated structure factor amplitudes, respectively. Summation includes all reflections used in the refinement.

c Free R factor = ∑| |*F*_*obs*_|-|*F*_*calc*_| |/∑|*F*_*obs*_|, evaluated for a randomly chosen subset of 5% of the diffraction data not included in the refinement.

d Root-mean square-deviation from ideal values.

e Categories were defined by Molprobity.

**Figure 1:**
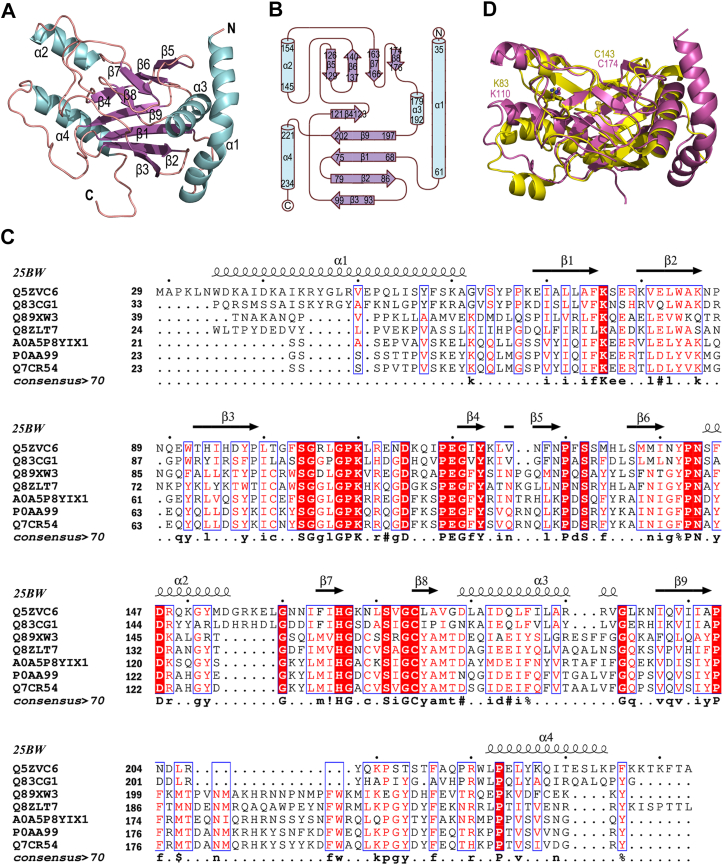
**Overall structure of Lpg1514**. *A*, cartoon representation of the overall fold of Lpg1514ΔN29. *B*, topology diagram of Lpg1514, with α-helices and β-strands shown as *cylinders* and *arrows*, respectively. *C*, sequence alignment of Lpg1514 with representative YafK family members. The sequence of Lpg1514 (Q5ZVC6, *L. pneumophila*, PDB: 25BW) was aligned with homologs from *Coxiella burnetii* (Q83CG1), *Bradyrhizobium diazoefficiens* (Q89XW3), *Salmonella typhimurium* (Q8ZLT7), *Yersinia pestis* (A0A5P8YIX1), *Escherichia coli* (DpaA, P0AA99), and *Salmonella typhimurium* (Q7CR54). Strictly conserved residues are highlighted with *red boxes*, and α-helices and β-strands are indicated by Greek letters α and β. *D*, structural superposition of Lpg1514 with DpaA (PDB: 8IKR), showing the side chains of Cys143, Lys83 (DpaA), and Cys174, Lys110 (Lpg1514) as *sticks*.

Lpg1514 adopts a compact fold composed of two mixed β-sheets (β4–β9–β1–β2–β3 and β5–β8) flanked by four α-helices (Fig. 1, *A*–*B*). A pronounced surface-exposed groove is formed by the β5–β8 sheet, the connecting loop regions, and the loop linking β3 and β4. This groove, with an estimated volume of approximately 330 Å^3^, defines a putative substrate-binding pocket (Figs. 1*A*, and S1*A*). Structure-guided sequence alignment indicates that residues lining this pocket are relatively conserved among YafK subfamily proteins (Fig. 1*C*), suggesting a shared functional role.

A structural similarity search using DALI identified DpaA from *Escherichia coli* (PDB: 8IKR), a well-characterized YafK protein, as the closest homolog. Structural superposition and sequence comparison reveal a high degree of similarity in the overall fold and in the architecture of the putative substrate-binding pocket (Fig. 1, *C*–*D*). Two residues essential for DpaA function, Cys143 and Lys83, are conserved in Lpg1514 as Cys174 and Lys110, respectively. In DpaA, substitution of these residues abolishes enzymatic activity, supporting their functional importance. To evaluate whether Lpg1514 retains structural features compatible with the canonical amidase function of YafK proteins, we performed docking analysis using γ-D-Glu-mDAP and L-Lys, which represent key components of the Braun’s lipoprotein–peptidoglycan linkage substrate recognized by DpaA-family amidases. Both molecules could be accommodated within the conserved substrate-binding pocket in catalytically plausible conformations, with the conserved cysteine residue positioned appropriately for nucleophilic attack (Fig. S1, *B*–*C*).

Taken together, these structural features indicate that Lpg1514 shares the conserved architecture of YafK proteins and is likely to possess a similar catalytic framework for amide bond hydrolysis.

### Lpg1514 contributes to β-lactam tolerance in *L. pneumophila*

**Figure 2:**
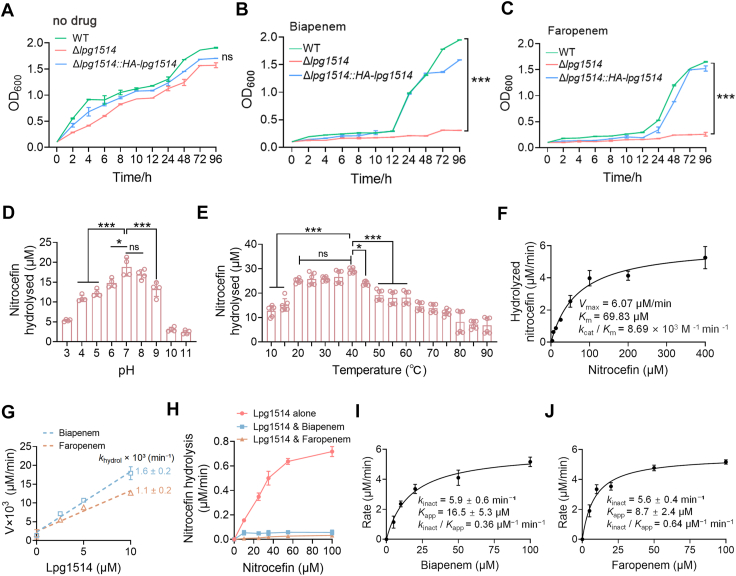
**Lpg1514 contributes to β-lactam tolerance and exhibits β-lactam–hydrolyzing activity.***A*, growth of *Lp02*, *Lp02*-*Δlpg1514* and the complemented strain (*Lp02-Δ1514::HA-lpg1514*) in AYE medium at 37 °C over 96 h in the absence of β-lactam antibiotics. *B*–*C*, growth of the indicated strains in the presence of biapenem at 1 μg/ml (*B*) or faropenem at 0.5 μg/ml (*C*). Bacterial growth was monitored by measuring OD_600_. *D*, effect of pH on nitrocefin hydrolysis by Lpg1514. *E*, Effect of temperature on nitrocefin hydrolysis by Lpg1514. *F*, Enzymatic activity of Lpg1514 toward nitrocefin at pH 7.0 and 25 °C. *G*, Apparent hydrolysis rates of biapenem (*blue*) and faropenem (*orange*) by Lpg1514. *H*, Residual nitrocefin hydrolysis activity following preincubation of Lpg1514 with biapenem or faropenem. *I* and *J*, time-dependent effects of biapenem (*I*) and faropenem (*J*) on Lpg1514 activity. Data are presented as mean ± SD from at least three independent experiments. Statistical significance was assessed by one-way ANOVA for panels B and C and by two-way ANOVA for *panels D* and *E*. ns, not significant (*p* > 0.05); ∗*p* < 0.05; ∗∗*p* < 0.01; ∗∗∗*p* < 0.001.

To evaluate the physiological role of Lpg1514, we compared the growth of wild-type and Δ*lpg1514* strains. Under standard culture conditions, deletion of *lpg1514* had no detectable effect on bacterial growth, indicating that Lpg1514 is not required for basal proliferation (Fig. 2*A*). In contrast, under β-lactam stress, including exposure to faropenem (0.5 μg/ml) and biapenem (1 μg/ml), the Δ*lpg1514* strain exhibited reduced growth compared with the wild type. This phenotype was restored upon complementation with wild-type *lpg1514*, demonstrating that Lpg1514 contributes to β-lactam tolerance in *L. pneumophila* (Fig. 2, *B*–*C*).

### Lpg1514 exhibits β-lactam hydrolysis activity

To determine whether Lpg1514 can directly interact with β-lactam substrates, its enzymatic activity was assessed using the chromogenic cephalosporin nitrocefin. Lpg1514 catalyzed nitrocefin hydrolysis, as indicated by an increase in absorbance at 486 nm, demonstrating that the protein possesses β-lactam–hydrolyzing activity. Biochemical characterization showed that Lpg1514 exhibits maximal activity at 40 °C and pH 7.0 (Fig. 2, *D*–*E*). Steady-state kinetic analysis indicated that nitrocefin hydrolysis follows Michaelis–Menten behavior, with a *V*_max_ of 6.07 μM min^−1^ and a *K*_m_ of 69.83 μM. Based on the enzyme concentration used in the assay, the catalytic efficiency (*k*_cat_/*K*_m_) was calculated to be 8.69 × 10^3^ M^−1^ min^−1^, indicating modest catalytic activity (Fig. 2*F*).

To further examine substrate specificity, the carbapenem antibiotics faropenem and biapenem were tested. Detectable enzyme-dependent hydrolysis of both compounds was observed after incubation of 50 μM substrate with increasing concentrations of Lpg1514 for 24 h, suggesting slow turnover (Fig. 2*G*). Consistent with this observation, pre-incubation of Lpg1514 with either antibiotic markedly reduced subsequent nitrocefin hydrolysis (Fig. 2*H*), indicating that these substrates form relatively stable complexes with the enzyme. To further characterize this interaction, inactivation kinetics were analyzed. The apparent second-order rate constants (*k*_inact_/*K*_app_) were determined to be 0.36 μM^−1^ min^−1^ for biapenem and 0.64 μM^−1^ min^−1^ for faropenem (Fig. 2, *I*–J), consistent with slow processing and prolonged enzyme–substrate residence time.

Together, these results indicate that Lpg1514 is capable of β-lactam hydrolysis, but with modest catalytic efficiency and slow turnover, particularly for carbapenem substrates.

### Ser132 is required for β-lactam hydrolysis, whereas Cys174 is dispensable

To gain insight into the catalytic mechanism, molecular docking of nitrocefin was performed using the Lpg1514ΔN29 structure. The model suggests a plausible binding mode in which the β-lactam ring is positioned near Ser132 and His166 within the substrate-binding pocket (Fig. S2*A*). In both the Lpg1514ΔN29 and Lpg1514ΔN29^C174A^ structures, electron density is observed in the vicinity of Ser132 (Fig. S2*B*), consistent with a role in ligand interaction.

**Figure 3:**
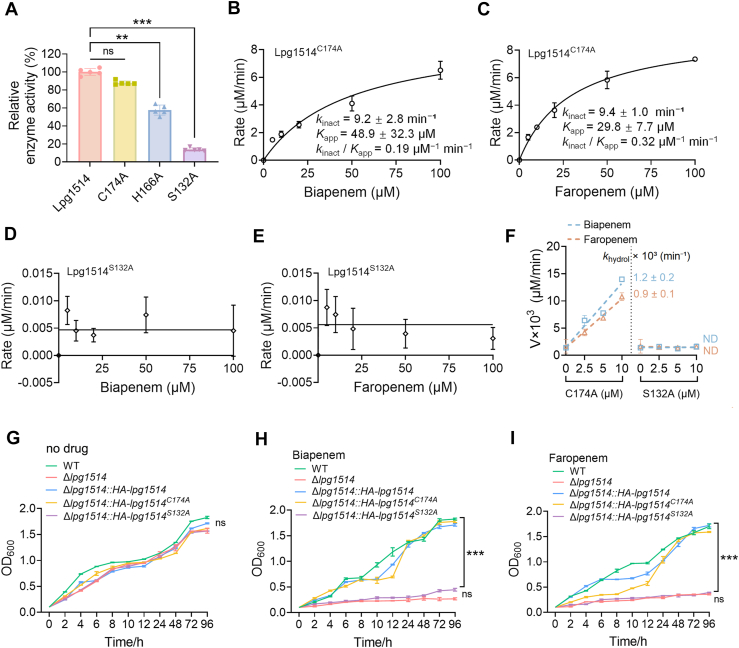
**Functional analysis of Lpg1514 variants.***A*, nitrocefin hydrolysis activity of wild-type Lpg1514 and its variants (C174A, H166A, and S132A). *B*–*E*, effects of biapenem (*B* and *D*) and faropenem (*C* and *E*) on the activity of Lpg1514^C174A^ (*B* and *C*) and Lpg1514^S132A^ (*D* and *E*). *F*, apparent hydrolysis rates of biapenem (*blue*) and faropenem (*orange*) by Lpg1514 variants. The intercept at enzyme concentration = 0 corresponds to the spontaneous hydrolysis rate of the carbapenem substrate. ND, not detected. *G*–*I*, growth of *L. pneumophila* strains expressing wild-type or mutant Lpg1514 in AYE medium at 37 °C over 96 h. (*G*) No antibiotic control. (*H* and *I*) Growth in the presence of biapenem (*H*) or faropenem (*I*). Data represent mean ± SD from at least three independent experiments. Statistical significance was determined using one-way ANOVA (*G*–*I*) or two-way ANOVA (*A*). ns, not significant (*p* > 0.05); ∗*p* < 0.05; ∗∗*p* < 0.01; ∗∗∗*p* < 0.001.

To test the functional importance of these residues, site-directed mutagenesis was performed. Substitution of the conserved cysteine (C174A), which is essential for amidase activity in other YafK proteins such as DpaA, did not significantly affect nitrocefin hydrolysis (Fig. 3*A*). A modest reduction in binding affinity toward carbapenem substrates was observed for the C174A mutant (Fig. 3, *B*–*C*). In contrast, substitution of Ser132 (S132A) abolished detectable activity (Fig. 3*A*), indicating that this residue is required for β-lactam hydrolysis. No detectable carbapenem-dependent signal was observed for the S132A mutant under the assay conditions, suggesting that Ser132 is required for productive interaction with carbapenem substrates (Fig. 3, *D*–*E*). Prolonged incubation assays (24 h) revealed that the C174A mutant retained detectable hydrolytic activity, whereas the S132A mutant showed no measurable activity (Fig. 3*F*).

Mutation of His166 resulted in a substantial reduction in activity (Fig. 3*A*), suggesting that it contributes to the hydrolytic process. The pKa of His166 was calculated using PROPKA (16), yielding a value of 5.26. This lower pKa, relative to typical histidine residues, is consistent with His166 being predominantly deprotonated under physiological conditions, suggesting it may facilitate proton transfer, potentially activating Ser132 during catalysis. However, direct evidence for this mechanism remains to be obtained.

Together, these results indicate that β-lactam hydrolysis by Lpg1514 requires Ser132, with His166 supporting this activity, while the conserved cysteine (Cys174) is dispensable.

To evaluate the physiological relevance of these findings, complementation experiments were conducted with the Δ*lpg1514* strain. Under standard growth conditions, all strains, including wild-type, mutants, and complemented strains, grew similarly (Fig. 3*G*). However, under β-lactam stress, expression of wild-type Lpg1514 or the C174A mutant restored bacterial growth, whereas the S132A variant did not (Fig. 3, *H*–*I*). These results establish a direct link between Ser132-dependent enzymatic activity and β-lactam tolerance in *L. pneumophila in vivo*.

## Discussion

In this study, we identify Lpg1514, a YafK family protein from *L. pneumophila*, as a contributor to β-lactam tolerance with detectable β-lactam hydrolysis activity. Structural, biochemical and genetic analysis reveal that this activity is distinct from the mechanisms employed by previously characterized YafK amidases and canonical YkuD L,D-transpeptidases. Members of YkuD superfamily, particularly L,D-transpeptidases, typically utilize a catalytic cysteine to mediate peptidoglycan cross-linking and interact with β-lactam antibiotics *via* covalent acylation (6, 14, 17). In contrast, YafK subfamily proteins have been functionally reassigned as L,D-meso-diaminopimelate protein amide hydrolases, where the conserved cysteine is essential for cleaving the Braun’s lipoprotein–peptidoglycan linkage (10, 12). Lpg1514 retains the conserved structural features and active-site architecture typical of YafK proteins, including the catalytic cysteine (Cys174), which is implicated in amidase activity. Although amidase activity was not directly assessed in this study, docking analyses using substrate components of Braun’s lipoprotein–peptidoglycan linkage suggest that Lpg1514 retains structural features compatible with the substrate-recognition mode of characterized YafK amidases. However, our data show that Cys174 is dispensable for β-lactam hydrolysis, indicating that this activity is mechanistically separable from the cysteine-dependent reactions described for both YafK amidases and YkuD enzymes. This distinction suggests that Lpg1514 may harbor multiple functional capabilities within a single structural scaffold.

A central finding of this study is the identification of Ser132 as a key residue required for β-lactam hydrolysis. While Ser132 is not part of the canonical YafK signature motif, it is conserved among YafK homologs. Structural analysis and docking studies position Ser132 in proximity to the β-lactam ring, and mutation of this residue abolishes enzymatic activity, underscoring its functional importance. Additionally, His166, located within the substrate-binding pocket, contributes to the activity. The calculated pKa of His166 suggests it may facilitate proton transfer, potentially activating Ser132 during catalysis. Unlike classical serine β-lactamases, where the catalytic serine is embedded within a conserved sequence motif and supported by an extensive catalytic network (18, 19), Ser132 in Lpg1514 is located outside the canonical YafK active site. This unique structural context implies that Lpg1514 employs a distinct catalytic strategy that may not involve a fully optimized catalytic triad.

Lpg1514 exhibits relatively low catalytic efficiency toward β-lactam substrates, which are hydrolyzed only after prolonged incubation. Pre-incubation with these carbapenems reduces subsequent nitrocefin hydrolysis, indicating stable enzyme-substrate interactions with slow turnover. This suggests that the activity of Lpg1514 may represent a latent or promiscuous catalytic capability, reflecting evolutionary plasticity within the YafK scaffold that allows for adaptation to environmental challenges, such as antibiotic exposure. This behavior resembles the interactions of L,D-transpeptidases from *Mycobacterium tuberculosis*, which also form long-lived complexes with β-lactam antibiotics (15). However, in those systems, substrate interaction is mediated by a catalytic cysteine residue, while in Lpg1514, the conserved cysteine is not involved in β-lactam hydrolysis. These differences indicate that, despite the slow turnover kinetics observed in both systems, the molecular mechanisms underlying the interactions with β-lactams are distinct. Notably, this activity does not appear to extend broadly across β-lactam subclasses. Representative penicillins and cephalosporins neither affected Lpg1514 activity in competition assays nor produced detectable Lpg1514-dependent phenotypes (Fig. S3). These observations suggest that Lpg1514 is unlikely to function as a broad-spectrum β-lactamase and instead displays a degree of substrate preference toward carbapenem-class compounds.

The identification of a serine-dependent β-lactam hydrolysis activity in Lpg1514 suggests that YafK proteins may possess broader functional capabilities than previously recognized. The coexistence of the conserved cysteine (associated with amidase activity) and the conserved serine (required for β-lactam hydrolysis in this study) hints at functional partitioning within the YafK scaffold. Given that Ser132 and residues forming the substrate-binding pocket are conserved across YafK homologs, we examined several representative members of the YafK subfamily. All tested homologs exhibited detectable nitrocefin hydrolysis activity, and mutational analysis of *E. coli* DpaA revealed residue requirements similar to those observed for Lpg1514 (Fig. S4). These findings suggest that the serine-dependent β-lactam–interacting mechanism identified here may be conserved within the YafK family rather than representing a unique property of Lpg1514.

Bacterial growth assays demonstrate that Lpg1514 contributes to β-lactam tolerance in *L. pneumophila* in a Ser132-dependent manner, linking the observed enzymatic activity to a physiological phenotype. However, the magnitude of this effect is moderate, suggesting that Lpg1514 is likely one of several factors contributing to antibiotic tolerance. The relatively low catalytic efficiency and slow turnover observed for Lpg1514 imply that its primary role may not be direct antibiotic degradation but rather transient interaction with β-lactams, contributing to tolerance rather than resistance.

Two limitations of this study should be noted. First, although docking analysis supports the possibility that Lpg1514 retains substrate-recognition features associated with DpaA-like amidases, direct biochemical validation will be required to establish the amidase activity. Second, the catalytic mechanism of β-lactam hydrolysis has not been fully resolved, particularly regarding reaction intermediates.

In conclusion, our results demonstrate that Lpg1514, a YafK family protein, exhibits β-lactam hydrolysis activity that relies on a conserved serine residue and operates independently of the canonical cysteine. This activity may represent a latent or promiscuous catalytic function embedded within the YafK scaffold, reflecting evolutionary flexibility that allows the protein to adapt to antibiotic pressure.

## Experimental procedures

Full experimental procedures can be found in the Supporting Information.

## Data availability

The structural factors and coordinates have been deposited in the Protein Data Bank under the accession codes 25BW and 25BY. The original data for this manuscript can be obtained from the corresponding author on reasonable request.

## Supporting information

This article contains supporting information (20, 21, 22, 23, 24, 25, 26, 27, 28, 29, 30, 31, 32, 33, 34, 35, 36, 37).

## Conflict of interest

The authors declare that they have no conflicts of interest with the contents of this article.
