# Supplementary material for: A YafK family protein from Legionella pneumophila exhibits serine-dependent β-lactam hydrolysis activity
Source: J Biol Chem. 2026 Jun 23;302(8):113293. doi: 10.1016/j.jbc.2026.113293 (PMC13400389; doi:10.1016/j.jbc.2026.113293)
Supplement: Supplementary Material [file mmc1.pdf]

Supporting Information for

A YafK family protein from *Legionella pneumophila* exhibits serine-dependent β-lactam hydrolysis activity

Wenwen Xu [a], Yuan Cao [a], Liangsan Cao [a], ChangYong Xiao [a], Wenjie Sun [a], Zhenhuang Ge [b], Weiqiang Wang [a]*, Jiajia Gao [a]*, Honghua Ge [a]*

[a] School of Life Sciences and Medical Engineering, Anhui University, Hefei, 230601, P. R. China
[b] School of Life Sciences, Sun Yat-sen University, Guangzhou, 510275, P.R.China
* For correspondence: Weiqiang Wang, weiqiang.wang@ahu.edu.cn; Jiajia Gao, 23748@ahu.edu.cn; Honghua Ge, hhge@ahu.edu.cn.

Experimental procedures

Protein production and purification

The coding sequence of Lpg1514 lacking the N-terminal 29 residues (Lpg1514ΔN29) and its variants were cloned into a modified p22 expression vector encoding an N-terminal 6×His-tag. Recombinant plasmids were transformed into *Escherichia coli* strain Rosetta. Cells were grown in Luria–Bertani (LB) medium supplemented with 100 µg/mL ampicillin at 37°C to an OD_{600} of 0.8–1.0, and protein expression was induced with 0.2 mM IPTG. Cells were further incubated at 16°C for 20 h before harvesting by centrifugation (5,000×g, 10 min, 4°C). Cell pellets were resuspended in lysis buffer (20 mM Tris-HCl, pH 8.0, 200 mM NaCl) and lysed by sonication on ice. The lysates were clarified by centrifugation (15,000 × g, 30 min, 4°C). The supernatant was applied to a Ni^{2}-charged chelating Sepharose column pre-equilibrated with lysis buffer. After washing with buffer containing 50 mM imidazole, bound proteins were eluted with buffer supplemented with 500 mM imidazole. The eluate was further purified by size-exclusion chromatography using a HiLoad 16/60 Superdex 200 column equilibrated in 20 mM Tris-HCl (pH 8.0) and 200 mM NaCl. Purified proteins were concentrated and stored in the same buffer. Protein concentrations were determined using the Bradford assay with bovine serum albumin as a standard.

Genes encoding *Escherichia coli* MG1655 DpaA and *Salmonella enterica* serovar Typhimurium Q7CR54 were amplified by PCR from genomic DNA and cloned into expression vectors. Genes encoding *Coxiella burnetii* Q83CG1 and *Yersinia pestis* A0A5P8YIX1 were synthesized and subcloned into the same expression system. All recombinant proteins were expressed in *E. coli* and purified using the same procedure as described above for Lpg1514. Purified proteins were analyzed by SDS-PAGE and visualized by Coomassie Brilliant Blue staining.

Crystallization and data collection

Crystallization trials were conducted using Crystal Screen, Crystal Screen 2, and PEG/Ion Screen kits (Hampton Research) at 287 K by the hanging-drop vapor-diffusion method. Each drop contained 1 µL of protein solution (10–30 mg/mL) and an equal volume of reservoir solution, equilibrated against 200 µL of reservoir solution. Optimization experiments included varying precipitant and protein concentrations and testing additives. Crystals of Lpg1514ΔN29 and the C174A variant were obtained at 14°C using a reservoir solution containing 0.2 M ammonium sulfate, 0.1 M sodium acetate (pH 4.5), and 30% (w/v) polyethylene glycol monomethyl ether 2000. Crystals were cryoprotected by brief transfer into reservoir solution supplemented with 20% (v/v) glycerol and flash-cooled in liquid nitrogen.

Diffraction data were collected at the BL17U and BL10U2 beamlines of the Shanghai Synchrotron Radiation Facility. Data were processed and scaled using XDS (20) in combination with programs from the CCP4 suite (21). Data collection statistics are summarized in Table 1.

Structure determination and analysis

The Lpg1514ΔN29 structure was solved by molecular replacement using PHASER (22) with an AlphaFold-predicted model as the search template (23). The structure of the C174A variant was solved using the refined Lpg1514ΔN29 model as the search model. Model building was performed using Coot (24), and refinement were carried out using REFMAC5 (25) and PHENIX (26). Model quality was evaluated with MolProbity (27). Refinement statistics are listed in Table 1. Coordinates and structure factors have been deposited in the Protein Data Bank under accession codes 25BW and 25BY.

Subfamily classification was obtained from the PANTHER (Protein ANalysis Through Evolutionary Relationships) database (28). Multiple sequence alignments were generated with Clustal Omega (29), and structure-based sequence alignments were prepared using ESPript (30). Structural homologs were identified using the DALI server (31). Protein–substrate docking was conducted with CB-Dock2 (32). Topology diagrams were generated with Topdraw (33), and all structural figures were created using PyMOL software (Schrödinger, LLC).

Enzymatic activity assays

Enzymatic activity of Lpg1514 was monitored using the chromogenic β-lactam substrate nitrocefin. Reactions (100 μL) were carried out in 100 mM buffer at the indicated pH values, containing enzyme and substrate at appropriate concentrations. Hydrolysis of nitrocefin was monitored by measuring the increase in absorbance at 486 nm using a molar extinction coefficient of 20,500 $M^{-1}\cdot cm^{-1}$.

For pH dependence assays, reactions were performed using sodium acetate buffer (pH 3.0–6.0), sodium phosphate buffer (pH 7.0–9.0), or glycine–NaOH buffer (pH 9.0–11.0). Temperature dependence was assessed over a range of 10–90°C. For kinetic measurements, reactions were conducted at 25°C in 100 mM sodium phosphate buffer (pH 7.0). Initial rates were determined from the linear phase of the reaction progress curves. Kinetic parameters were obtained by fitting the data to the Michaelis–Menten equation using nonlinear regression in GraphPad Prism (version 6.01, USA).

Effect of β-lactam compounds on enzymatic activity

To assess the inhibitory activity of β-lactam antibiotics on Lpg1514, reactions were performed using nitrocefin as the substrate. In 100 mM sodium phosphate buffer (pH 7.0), 10 μM Lpg1514 was preincubated with 50 μM of each β-lactam at 25°C for 2 h. After preincubation, nitrocefin was added at a range of concentrations to the enzyme–inhibitor mixtures and incubated at 25°C for 50 min. Hydrolysis of nitrocefin was monitored by measuring absorbance at 486 nm, and reaction rates (μM/min) were calculated according to Beer's law ($\varepsilon = 20{,}500\ M^{-1}\cdot cm^{-1}$ for nitrocefin; L = 0.5 cm).

Determination of Catalytic Constants

Acylation kinetics were measured using a microplate reader (SpectraMax iD_3) by monitoring the decrease in absorbance associated with β-lactam ring opening. The kinetics of acyl-enzyme complex formation were determined by mixing 10 μM Lpg1514 with increasing concentrations of each β-lactam antibiotic in 100 mM sodium

phosphate buffer (pH 7.0) at 10°C. Reactions were performed at 10°C to reduce reaction rates, similar to previously reported studies employing stopped-flow or fluorescence-based approaches (34). The rate of acyl-enzyme complex formation was calculated from the observed absorbance decrease. The λ_{max} of the antibiotics was determined spectrophotometrically: 300 nm for biapenem and 306 nm for faropenem. Corresponding molar extinction coefficients were 9846 $M^{-1}\cdot cm^{-1}$ for biapenem and 6980 $M^{-1}\cdot cm^{-1}$ for faropenem. Kinetic parameters, including k_{inact} and K_{app}, were obtained by fitting the data to a nonlinear regression model.

Kinetics of acyl-enzyme hydrolysis

Hydrolysis rates of acylenzyme complexes were assessed by incubating 50 μM β-lactam antibiotics with varying concentrations of Lpg1514 (0, 2.5, 5 and 10 μM) at 25°C in 100 mM sodium phosphate buffer (pH 7.0). The progress of β-lactam cleavage was monitored by absorbance at 300 nm for biapenem and 306 nm for faropenem using a microplate reader (SpectraMax iD3). Hydrolysis velocities (V) were calculated and plotted against enzyme concentration, and enzyme turnover was calculated from the slope of the hydrolysis velocity versus enzyme concentration plot.

Bacterial strains and construction of knockout strains

The bacterial strains used in this study are listed in Table S1. *E. coli* strains were routinely cultured in LB medium, supplemented with antibiotics when required. All *Legionella pneumophila* strains were derived from the Philadelphia-1 *Lp02* background and grown on ACES-buffered charcoal yeast extract (CYE) medium. Where required, thymidine (0.1 mg/mL) was added for auxotrophic strains. In-frame deletion mutants of *lpg1514* were generated via allelic exchange using the suicide vector pK18mobsacB. Deletion constructs were designed to replace the target gene with a 30–amino acids sequence comprising the N- and C-terminal 15 residues. The recombinant plasmid pK18mobsacB-Δ*lpg1514* was introduced into *Lp02* and *Lp02*-Δ*lpg1514* strains by electroporation (2.0 kV, 200 Ω, 5 ms). Transformants were initially selected on CYET agar containing kanamycin, followed by counter-selection on CYET agar supplemented with 10% sucrose at 37°C for 3 days. Deletion mutants were confirmed by PCR and DNA sequencing.

Genetic complementation

The coding sequences of Lpg1514 and its variants were cloned into the RSF1010-based plasmids pBBR1MCS-2-HA. The resulting plasmids were introduced into *Lp02*-Δ*lpg1514* strain by electroporation. Complemented strains were selected on CYET agar with kanamycin. Expression of the complemented proteins was verified by immunoblotting using an anti-HA antibody (Figure S5). A list of plasmids and primers used in this study is provided in Tables S2 and S3.

Minimum inhibitory concentration assay

The minimum inhibitory concentration (MIC) of various antibiotics were determined using the broth microdilution method (35), according to Clinical and Laboratory Standards Institute (36). The MIC results are shown in Table S4.

Bacterial growth assays

Bacterial growth assays were conducted by inoculating *L. pneumophila* into AYET medium containing the appropriate antibiotics and incubating cultures at 37°C with shaking at 220 rpm. Growth was monitored by measuring OD_{600} at 2-h intervals during the exponential phase. In the bacterial growth assay, the test concentration was set at half the MIC determined for the Δ*lpg1514* strain.

Western blotting

L. pneumophila cells were lysed in RIPA buffer (Beyotime, P0013B, CHN) supplemented with 1 mM PMSF and incubated on ice at 4°C for 30 min. The lysates were centrifuged, and the resulting supernatants were combined with SDS loading buffer and boiled for 5 min. Proteins were then separated by SDS–PAGE, transferred onto PVDF membranes, and immunoblotted with antibodies against HA and ICDH (Table S5). The anti-ICDH antibody was used as a cytoplasmic marker and loading control to verify sample integrity and exclude nonspecific lysis.

Statistical analysis

Data are shown as mean ± standard deviation (SD) from at least three independent experiments and were analyzed using GraphPad Prism (version 6.01, USA). Statistical significance was determined using one-way or two-way ANOVA. P values < 0.05 were considered statistically significant.

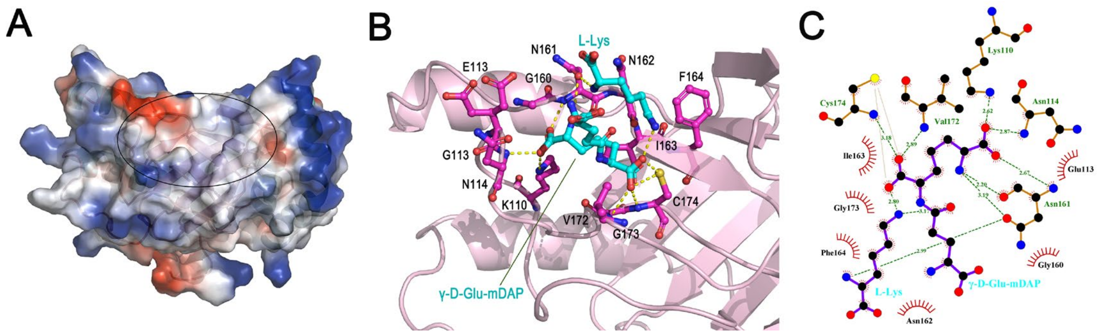

Figure S1. Structural modeling of putative amidase-substrate recognition by Lpg1514. ***A***, Electrostatic surface representation of Lpg1514ΔN29 shown in the same orientation as Fig. 1A. Positive and negative electrostatic potentials are colored blue and red, respectively. The putative substrate-binding groove is indicated. ***B***, Docking models of γ-D-Glu-mDAP and L-Lys within the substrate-binding pocket of Lpg1514. Substrates are shown as cyan sticks, and residues predicted to participate in substrate recognition are shown as magenta sticks. ***C***, LigPlot representations of predicted interactions between Lpg1514 and the docked substrates. Hydrogen bonds and hydrophobic contacts were generated using LigPlot (37) to visualize potential substrate-recognition networks within the conserved binding pocket.

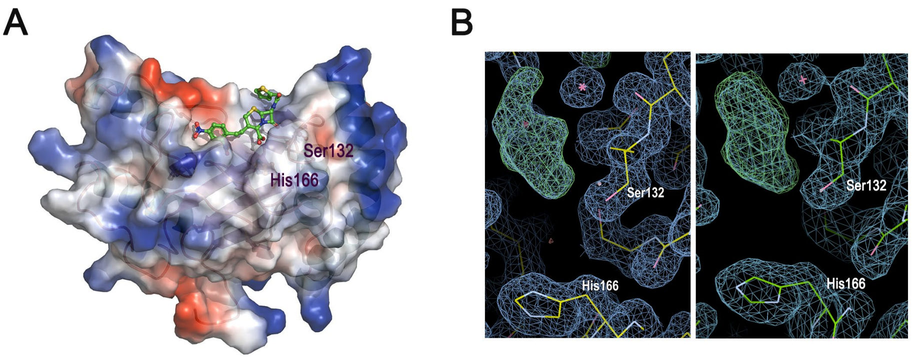

Figure S2. Structural features associated with substrate recognition and catalysis in Lpg1514. ***A***, Docking model of the Lpg1514–nitrocefin complex displayed on the electrostatic surface, with nitrocefin shown as sticks. ***B***, Distinct electron density near Ser132 in the Lpg1514ΔN29 (left) and Lpg1514ΔN29^{C174A} (right). Blue meshes (σ = 1.0) and green meshes (σ = 3.5) represent the 2Fo-Fc map and the Fo-Fc omit map, respectively.

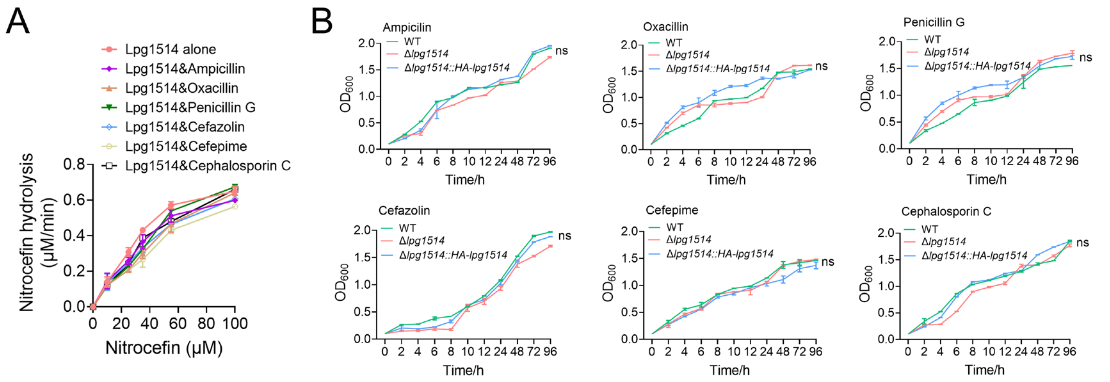

Figure S3. Assessment of Lpg1514 interactions with non-carbapenem β-lactam antibiotics. ***A***, Residual nitrocefin hydrolysis activity following preincubation with representative β-lactam antibiotics. Purified Lpg1514 was preincubated with ampicillin, oxacillin, penicillin G, cefazolin, cefepime, or cephalosporin C prior to measurement of nitrocefin hydrolysis under standard assay conditions. Residual activity was normalized to that of the untreated enzyme control. ***B***, Growth of *Legionella pneumophila* strains in the presence of non-carbapenem β-lactam antibiotics. *Lp02*, *Lp02-Δlpg1514* and the complemented strain (*Lp02-Δ1514::HA-lpg1514*) were cultured in the presence of the indicated antibiotics, and bacterial growth was monitored by measuring OD600 at the indicated time points.

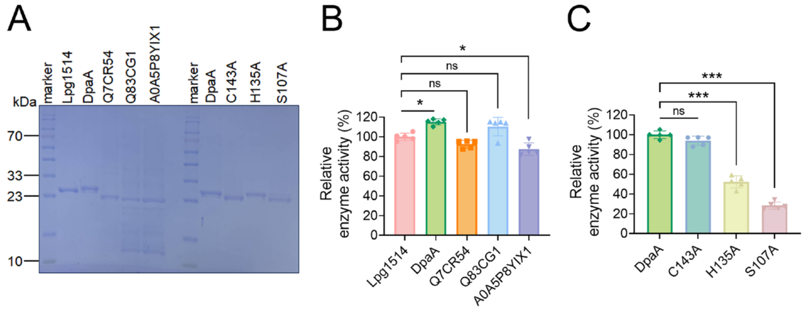

Figure S4. Conservation of nitrocefin hydrolysis activity among YafK homologs. ***A***, SDS-PAGE analysis of purified recombinant proteins. DpaA (*Escherichia coli* MG1655), Q7CR54 (*Salmonella typhimurium*), Q83CG1 (*Coxiella burnetii*), A0A5P8YIX1 (*Yersinia pestis*) and DpaA variants (C143A, H135A and S107A) were expressed in E. coli and purified by Ni2+-affinity and size-exclusion chromatography. Molecular mass markers (kDa) are indicated. ***B***, Nitrocefin hydrolysis activity of representative YafK homologs. Purified proteins (10 μM) were incubated with nitrocefin (100 μM) at 25°C for 10 min, and hydrolysis was monitored by measuring absorbance changes at 486 nm. Activities were normalized to that of Lpg1514. ***C***, Nitrocefin hydrolysis activity of DpaA variants. Wild-type DpaA and the indicated mutants were analyzed under the same conditions as in panel B. Activities were normalized to that of wild-type DpaA. Data are presented as mean ± SD from at least three independent experiments. Statistical significance was evaluated by one-way ANOVA. ns, not significant ($P > 0.05$); *$P < 0.05$, ***$P < 0.001$.

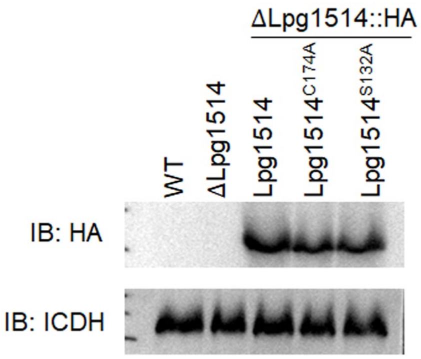

Fig. S5. Validation of Lpg1514 complementation strains. Expression of HA-tagged Lpg1514 and its variants was verified by immunoblotting using an anti-HA antibody. ICDH was used as internal reference.

Table S1. Bacterial strains used in this study.

Bacterial strains	Source	Country	Identifier Cat.
E. coli DH5α	TSINGKE	CHN	Cat: # TSC-C14
E. coli BL21(DE3) Rosetta	Sigama	GER	Cat: # 70954
L. pneumophila strain Lp02	A gift from Prof. Yongjun Lu (Sun Yat-sen University)		

Table S2. Plasmids used in this study.

Plasmids	Genotype	Purpose	Reference or source
p22	Cloning vector, Amp ^{r}	Cloning	Derived from pET-22b
p22-6His-Lpg1514ΔN29	Cloning vector encoding Lpg1514ΔN29, Amp ^{r}	Protein expression &purification	This study
p22-6His-Lpg1514ΔN29^{S132A}	Cloning vector encoding Lpg1514ΔN29 catalytic S132A mutant, Amp ^{r}	Protein expression &purification	This study
p22-6His-Lpg1514ΔN29^{H166A}	Cloning vector encoding Lpg1514ΔN29 catalytic H166A mutant, Amp ^{r}	Protein expression &purification	This study
p22-6His-Lpg1514ΔN29^{C174A}	Cloning vector encoding Lpg1514ΔN29 catalytic C174A mutant, Amp ^{r}	Protein expression &purification	This study
pK18mobsacB	Widely used gene knockout vector, Kan ^{r}	Gene knockout	
pK18mobsacB-Lpg1514	Gene knockout vector knocking out Lpg1514	Gene knockout	This study
pBBR1MCS	Widely used gene expression vector, Kan ^{r}	Gene expression	
pBBR1MCS-HA-Lpg1514	Gene expression vector expressing HA-Lpg1514, Kan ^{r}	Gene expression	This study
pBBR1MCS-HA-Lpg1514^{S132A}	Gene expression vector expressing HA-Lpg1514^{S132A}, Kan ^{r}	Gene expression	This study
pBBR1MCS-HA-Lpg1514^{C174A}	Gene expression vector expressing HA-Lpg1514^{S132A}, Kan ^{r}	Gene expression	This study
p22-DpaAΔN19-6His	Cloning vector encoding DpaAΔN19, Amp ^{r}	Protein expression	This study

Plasmids	Genotype	Purpose	Reference or source
		&purification	
p22-DpaAΔN19^{S107A}-6His	Cloning vector encoding DpaAΔN19 catalytic S107A mutant, Amp ^{r}	Protein expression &purification	This study
p22-DpaAΔN19^{H135A}-6His	Cloning vector encoding DpaAΔN19 catalytic H135A mutant, Amp ^{r}	Protein expression &purification	This study
p22-DpaAΔN19^{C143A}-6His	Cloning vector encoding DpaAΔN19 catalytic C143A mutant, Amp ^{r}	Protein expression &purification	This study
p22-Q7CR54$_{23\text{-}221}$-6His	Cloning vector encoding Q7CR54$_{23\text{-}221}$, Amp ^{r}	Protein expression &purification	This study
pET28a-Q83CG1ΔN32-6His	Cloning vector encoding Q83CG1ΔN32, Kan ^{r}	Protein expression &purification	This study
pET28a-A0A5P8YIX1$_{21\text{-}219}$-6His	Cloning vector encoding A0A5P8YIX1$_{21\text{-}219}$, Kan ^{r}	Protein expression &purification	This study

Table S3. Primers used in this study

ID	Name	Sequence (5' to 3')
1	HA-Lpg1514^{S132A} forw. (pBBR1MCS-HA-Lpg1514^{S132A})	TAATCCTTTTGCCAGCATGCACTTATCCATGATG
2	HA-Lpg1514^{S132A} rev. (pBBR1MCS-HA-Lpg1514^{S132A})	GATAAGTGCATGCTGGCAAAAGGATTAAAATTTA C
3	HA-Lpg1514^{C174A} forw. (pBBR1MCS-HA-Lpg1514^{C174A})	TGTAGGAGCTTTGGCTGTCGGTGACCTTG
4	HA-Lpg1514^{C174A} rev. (pBBR1MCS-HA-Lpg1514^{C174A})	ACAGCCAAAGCTCCTACAGATAAATTTTTTCC
5	6His-Lpg1514ΔN29 forw. (p22-6His-Lpg1514ΔN29)	GTCATCACCATCATCATCACGGATCCGCACCTAAG CTTAACTGGGATAAAG
6	6His-Lpg1514ΔN29 rev. (p22-6His-Lpg1514ΔN29)	CAGTGGTGGTGGTGGTGGTGCTCGAGTTAAGCAG TAAATTTAGTCTTTTTAAAGGG
7	C174A-Lpg1514ΔN29 forw. (p22-6His-Lpg1514ΔN29^{C174A})	TGTAGGAGCTTTGGCTGTCGGTGACCTTG
8	C174A-Lpg1514ΔN29 rev. (p22-6His-Lpg1514ΔN29^{C174A})	ACAGCCAAAGCTCCTACAGATAAATTTTTTCC
9	H166A-Lpg1514ΔN29 forw. (p22-6Hi-Lpg1514ΔN29^{H166A})	GGAAATAATATTTTTATTGCTGGAAAAAAT
10	H166A-Lpg1514ΔN29 rev. (p22-6His-Lpg1514ΔN29^{H166A})	TTTTCCAGCAATAAAAATATTATTTTCCTAACTC
11	S132A-Lpg1514ΔN29 forw. (p22-6His-Lpg1514ΔN29^{S132A})	TAATCCTTTTGCCAGCATGCACTTATCCATGATG
12	S132A-Lpg1514ΔN29 rev. (p22-6His-Lpg1514ΔN29^{S132A})	GATAAGTGCATGCTGGCAAAAGGATTAAAATTTA C
13	Lpg1514 P1 forw. (pK18mobSacB)	CAGGAAACAGCTATGACATGATTACGAATTCATAA AAACTAAATAGCAGAGCGTC
14	Lpg1514 P2 rev. (pK18mobSacB)	GTTTGAGTGATTCTGTAAAAAATCGTTTCATAGAC GCACTATAC
15	Lpg1514 P3 forw. (pK18mobSacB)	CTATGAAACGATTTTTACAGAATCACTCAAACCCT TTAAAAAGAC
16	Lpg1514 P4 rev. (pK18mobSacB)	GTAAAACGACGGCCAGTGCCAAGCTTGTGTTTAT TTGCCACATCAATGAAATAG
17	Lpg1514 Q forw. (pBBR1MCS-HA-Lpg1514)	CGATAAGCTTGATATCGAATTCATCGAAAACAGGT TGAATGGAAC
18	Lpg1514 Q rev. (pBBR1MCS-HA-Lpg1514)	CCTTGTAGTCCATACTATCAAATAATAAATATCAGA ACAATTTG
19	Lpg1514 HA forw. (pBBR1MCS-HA-Lpg1514)	ATTATTTGATAGTATGGACTACAAGGACGACGATG AC
20	Lpg1514 HA rev. (pBBR1MCS-HA-Lpg1514)	GTTACACACATGAATTCAGGCGCGCCTCCG
21	Lpg1514 HA forw. (pBBR1MCS-HA-Lpg1514)	GCGCCTGAATTCATGTGTGTAACTTACCAGTATAG TGC

ID	Name	Sequence (5' to 3')
22	Lpg1514 HB rev. (pBBR1MCS-HA-Lpg1514)	GATCCCCCGGGCTGCAGGAATTCTTAAGCAGTAA ATTTAGTCTTTTTAAAGGG
23	DpaAΔN19-6His forw. (p22-DpaAΔN19-6His)	AACTTTAAGAAGGAGATATACCATGGGCGGTTTG CTGGGCAGCAGTAGC
24	DpaAΔN19-6His rev. (p22-DpaAΔN19-6His)	TCAGTGGTGGTGGTGGTGGTGCTCGAGTTTTGCC TCGGGGAGCGTGTAGTTTG
25	S107A-DpaAΔN19 forw. (p22-DpaAΔN19^{S107A}-6His)	AACCAGACGCCCGTTACTACAAAGCG
26	S107A-DpaAΔN19 rev. (p22-DpaAΔN19^{S107A}-6His)	GTAACGGGCGTCTGGTTTTAACTGATTACG
27	H135A-DpaAΔN19 forw. (p22-DpaAΔN19^{H135A}-6His)	CTGATGATCGCCGGCGATTGTGTTTCCATC
28	H135A-DpaAΔN19 rev. (p22-DpaAΔN19^{H135A}-6His)	AATCGCCGGCGATCATCAGGTATTTCCC
29	C143A-DpaAΔN19 forw. (p22-DpaAΔN19^{C143A}-6His)	TCCATCGGCGCCTACGCAATGACCAATC
30	C143A-DpaAΔN19 rev. (p22-DpaAΔN19^{C143A}-6His)	CATTGCGTAGGCGCCGATGGAAACACAATC
31	Q7CR54$_{23-221}$-6His forw. p22-Q7CR54$_{23-221}$-6His	AACTTTAAGAAGGAGATATACCATGGGCAGCAGC AGCAGCCCCGTCAC
32	Q7CR54$_{23-221}$-6His rev. p22-Q7CR54$_{23-221}$-6His	TCAGTGGTGGTGGTGGTGGTGCTCGAGGTAACGT CCATCAACGATAGAAACAGTCGGCG

Table S4. MICs of various antibiotics against *Lp02* strains.

	MIC (μg/mL)		
**Antimicrobial**	***Lp02***	***Lp02-Δlpg1514***	***Lp02-Δlpg1514::HA-lpg1514***
Biapenem	8	2	4
Faropenem	16	1	4
Ampicillin	128	32	64
Oxacillin	128	64	128
Penicillin G	128	32	64
Cefazolin	256	16	128
Cefepime	8	1	4
Cephalosporin C	512	32	128

Table S5. Reagent and resource used in this study.

REAGENT or RESOURCE	SOURCE	IDENTIFIER Cat：
**Antibodies**		
Rabbit Polyclonal anti-HA	Proteintech	Cat: # 51064-2-AP
Rabbit Polyclonal-ICDH	Sigma-Aldrich	Cat: # ABS2090
HRP-conjugated Affinipure Goat Anti- Rabbit IgG(H+L)	Proteintech	Cat: # SA00001-2
